# An unusual case of a posterior mediastinal myelolipoma in a patient with mediterranean anemia

**DOI:** 10.11604/pamj.2018.31.58.17041

**Published:** 2018-09-27

**Authors:** Piergiorgio Muriana, Giampiero Negri

**Affiliations:** 1 Department of Thoracic Surgery, San Raffaele Scientific Institute, Milan, Italy

**Keywords:** Myelolipoma, mediastinal tumor, mediterranean anemia, differential diagnosis

## Image in medicine

A 34-year-old male was admitted at our Department for back pain and a right paravertebral mass at the chest x-ray. His medical history was positive for beta-thalassemia major with normal routinely blood tests. A contrast-enhanced chest computed-tomography showed a 26 x 15 mm right solid paravertebral round-shaped lesion at T8-T9 levels, with a small inner component of adipose tissue (Panel A). Contrast-enhanced magnetic resonance-imaging showed irregular low signal intensity in long TR sequences, with mild enhancement after contrast administration (Panel B, Panel C). Surgical excision of the lesion by means of video-assisted thoracic surgery was scheduled for both definitive diagnosis and therapeutic purpose. Histopathologic examination revealed the presence of adipocytes mixed with mature hematopoietic cells (Panel D). Differential diagnosis included mediastinal myelolipoma (MM) and extramedullary hematopoiesis (EH). In fact, both of them are composed of fat and hematopoietic tissue. Mediterranean anemia is a common finding in patients with EH. However, the presence of a single capsulated tumor and the absence of abnormal hematopoietic cells led to a final diagnosis of MM. The patient is currently alive without recurrence 23 months after surgery. Myelolipoma is a benign tumor usually arising in adrenal glands. Less than 50 cases of MM have been described in the literature to date. Many Authors currently support the role of a triggering condition (both metabolic, infectious and neoplastic), which might be responsible for the growth of ectopic adrenal or hematopoietic tissue. Patient's history, radiologic, and pathologic features must all be taken into due consideration in differential diagnosis between MM and EH.

**Figure 1:**
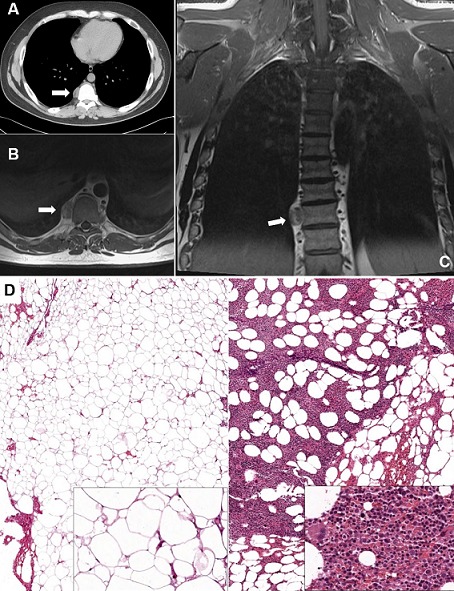
(A) axial chest CT-scan; (B) axial chest MRI; (C) coronal chest MRI; (D) microscopic specimen (stained with hematoxylin and eosin) of MM showing the presence of mature adipocytes (left), and hematopoietic tissue (right), consisting of erythroid and myeloid cells at different stages of differentiation. In addition, scattered megakaryocytes, rare lymphocytes and plasmacells; original magnification 40X, inset 200X

